# Characterization and Comparative Analysis of Gut Microbiomes in Fourteen Parrot Species

**DOI:** 10.3390/vetsci13020185

**Published:** 2026-02-12

**Authors:** Chanhyeok Park, Hyukjung Kim, Junhyeok Yoon, Aryung Nam

**Affiliations:** 1 Department of Stem Cell and Regenerative Biotechnology, Institute of Advanced Regenerative Science, Konkuk University, Seoul 05029, Republic of Korea; chpark0729@gmail.com (C.P.); mykhj102@gmail.com (H.K.); yoonjh0430@naver.com (J.Y.); 2 EPIOME Inc., Seoul 05029, Republic of Korea; 3 GIST InnoCORE AI-Nano Convergence Institute for Early Detection of Neurodegenerative Diseases, Gwangju Institute of Science and Technology, Gwangju 61005, Republic of Korea; 4 Department of Veterinary Internal Medicine, College of Veterinary Medicine, Konkuk University, Seoul 05029, Republic of Korea

**Keywords:** parrot gut microbiome, microbial diversity, next-generation sequencing, 16S rRNA sequencing, comparative analysis

## Abstract

Although the gut microbiome constitutes a key component of vertebrate physiology, comparative baseline data for companion birds, particularly parrots, remain limited. Therefore, this study profiled the fecal gut microbiota of 31 privately owned companion parrots representing 14 psittacine species maintained in indoor household environments for >6 months. Amplicons targeting the V3–V4 region of the 16S rRNA gene were sequenced, denoised into amplicon sequence variants using QIIME 2 with DADA2, and taxonomically assigned against the SILVA v132 database. Community composition was broadly dominated by *Firmicutes* and *Proteobacteria*, with recurrent detection of *Lactobacillus* across most samples, consistent with a potential core component of the gut microbiome of captive parrots. Taken together, this study provides an exploratory comparative snapshot of fecal gut microbiota across diverse companion parrot species and establishes baseline reference data for future research linking diet, husbandry practices, and health to microbiome variation, including longitudinal and wild–captive comparative investigations.

## Simple Summary

The gut microbiome is increasingly recognized as an important factor that influences animal health, digestion, and disease susceptibility. However, data on companion birds, particularly parrots, remains limited. This study analyzed the fecal gut microbiota of 31 parrots from 14 psittacine species using 16S rRNA gene sequencing. Although major bacterial groups such as *Firmicutes* and *Proteobacteria* were consistently present across all parrots, microbial diversity and community composition varied greatly among individuals, even within the same species. No clear species-specific microbial patterns were observed, suggesting that environmental factors, such as diet and husbandry, play a greater role in shaping the gut microbiome of parrots than host species. *Lactobacillus* was commonly detected across samples, indicating a potential core bacterial group in parrots. These findings provide foundational data on the gut microbiota of parrots and may inform future microbiome-based approaches to improve the health, nutrition, and management of captive parrots in veterinary practice.

## 1. Introduction

The gut microbiome has emerged as a central determinant of host physiology, shaping digestion, nutrient absorption, immune function, and behavior through complex host–microbe interactions [1,2,3]. Extensive microbiome studies in mammals, including humans, have revealed strong associations between microbial community structure and various health outcomes, ranging from metabolic disorders and immune regulation to neurological functions [4,5,6,7]. Studies in mice and humans have linked gut microbiome configuration to obesity and energy harvest [1,8], and controlled dietary interventions have been shown to rapidly alter human gut community structure and function [9]. Gut microbiome perturbations have also been implicated in metabolic dysregulation, such as glucose intolerance [10]. This expanding body of research underscores that the gut microbiome is a critical mediator of host health and disease.

Despite the ecological and economic significance of birds, the avian gut microbiome remains underexplored compared with that of mammals. Avian gut microbial communities are shaped by an interplay of host ecology and external conditions, with diet, habitat, seasonality, and captivity consistently identified as major drivers of community structure [11]. Birds exhibit distinctive gastrointestinal physiology and rapid food transit compared with mammals. Chickens and other poultry species have received considerable attention due to their agricultural importance, and studies have linked gut microbiota to growth efficiency, pathogen resistance, and feed utilization [12,13]. Although gut microbiome research in non-domesticated and companion birds, such as parrots, remains relatively limited, several studies have begun to characterize parrot-associated gut microbiota [14,15,16,17]. Parrots (order *Psittaciformes*) represent one of the most diverse avian lineages and are characterized by complex social behaviors, long lifespans, and broad dietary niches [18,19]. These ecological and dietary differences provide a useful framework for testing how feeding ecology shapes gut microbiome assembly, as diet-associated shifts in fecal gut microbiota have been reported [20,21]. Recent studies across diverse wild and managed avian systems indicate that gut microbiomes can be highly responsive to environmental change [14,22]. Consequently, captivity and standardized feeding regimes may alter microbial diversity and composition relative to wild conditions, complicating cross-study comparisons and underscoring the need for species- and context-specific baseline data for interpretation.

Previous studies have profiled the gut microbiota of various parrot species, including cockatiels and Alexandrine parakeets [20,21]. The present study conducted a comparative gut microbiota analysis of 14 psittacine species, comprising 31 parrots maintained under controlled household conditions, to characterize microbial community composition and assess both alpha and beta diversity.

## 2. Materials and Methods

### 2.1. Fecal Sample Collection

Fecal samples were collected from 31 parrots belonging to 14 psittacine species, including *Agapornis roseicollis* (*n* = 2), *Amazona aestiva* (*n* = 4), *Amazona ochrocephala* (*n* = 3), *Ara ararauna* (*n* = 3), *Cacatua alba* (*n* = 3), *Cacatua leadbeateri* (*n* = 1), *C. sulphurea* (*n* = 2), *Eclectus roratus* (*n* = 1), *Lorius chlorocercus* (*n* = 1), *Myiopsitta monachus* (*n* = 2), *Pionites leucogaster* (*n* = 1), *Poicephalus senegalus* (*n* = 2), *Psittacus erithacus* (*n* = 1), and *Pyrrhura molinae* (*n* = 5). All individuals were privately owned companion parrots acquired from commercial parrot breeders and had been maintained in indoor household environments for >6 months prior to sampling. Individual-level metadata were recorded, including estimated age (range: 1–4 years), health status (all birds were clinically healthy with no antibiotic administration within 3 months prior to sampling), and housing type (individual- or pair-housed indoor aviaries). All feeds were obtained from commercial suppliers and replaced daily. Fresh water was provided ad libitum. To obtain an accurate gut microbiota, the cages were cleaned, and feces were collected using a preservation kit (Noble Biosciences, Hwaseong, Republic of Korea). Finally, 31 fecal samples were stored in a deep freezer at 80 °C immediately after excretion.

### 2.2. DNA Extraction

DNA was extracted from fecal samples using an ARA MagNA DNA Isolation Kit (LAS, Gimpo, Republic of Korea), according to the manufacturer’s instructions. Briefly, 200 μL of each fecal sample was transferred to a 2 mL tube containing 20 uL (40 mg/mL) proteinase K and 0.3 mL PL1 lysis buffer (LAS). After rotation with vibration for 10 min, the samples were centrifuged to pellet debris. The matrix was lysed at room temperature at 12,000× *g* for 2 min. After centrifuging, 0.4 mL of the supernatant was mixed with 0.4 mL PB2 binding buffer (LAS) and 20 uL magnetic beads. The extracted DNA samples were stored at −20 °C until use.

### 2.3. Library Preparation and Sequencing

A 16S rRNA sequencing library was constructed targeting the V3–V4 hypervariable regions of the 16S rRNA gene. Polymerase chain reaction (PCR) and purification of PCR products were performed using KAPA HiFi HotStart ReadyMix (KAPA Biosystems, Wilmington, MA, USA) and ARAClean Beads (LAS), respectively. The initial PCR was performed with template DNA using region-specific primers compatible with MGI index and sequencing adapters (forward primer: 5′-GGCTCACAGAACGACATGGCTACGATCCGACTTCCTACGGGNGGCWGCAG-3′; reverse primer: 5′-TTGTCTTCCTAAGACCGCTTGGCCTCCGACTTGACTACHVGGGTATCTAATCC-3′). After magnetic bead-based purification of PCR products, the second PCR was performed using primers from an MGIEasy UDB Primers Adapter Kit A (MGI, Shenzhen, China) with a limited cycle. The purified PCR products were subsequently visualized by gel electrophoresis and quantified using a Qubit dsDNA HS Assay Kit (Invitrogen, Waltham, MA, USA) on a Qubit 4.0 fluorometer. Further circularization of the library was performed using the MGIEasy dual-barcode circularization module (MGI, Shenzhen, China). The pooled library was circularized at 37 °C for 30 min, followed by digestion at 37 °C for 30 min and cleanup of the circularization products. To prepare DNA nanoballs (DNB), the library was incubated at 30 °C for 15 min using the DNB enzyme. Finally, a Qubit ssDNA HS Assay Kit (Invitrogen) was used to quantify the library. The prepared DNB was sequenced using the MGIseq system (MGI) with 300 bp paired-end reads. Sequencing quality was verified by assessing specified quality control measures. This assessment was performed using FastQC and MultiQC [23].

### 2.4. Microbial 16S rRNA Gene Sequencing Data Analysis

To ensure the same total number of reads across samples and normalized microbial reads, rarefaction curves were generated to verify sufficient recovery of all existing operational taxonomic units. The microbial 16S rRNA sequencing data were processed using the QIIME 2 (2024.2) next-generation microbiome bioinformatics platform [24]. All input data were formatted as QIIME 2 artifacts, including details of the data types and sources. FASTQ reads were imported using import commands in the tools. Sequence quality control and feature table construction were performed using the DADA2 plugin in QIIME 2 [25]. After denoising, feature data were assigned using a pretrained Naïve Bayes classifier artifact from the scikit-learn library within the QIIME 2 pipeline. This classifier was trained against the SILVA v132 database, trimmed to include only V34 hypervariable regions, and pre-clustered at 99% sequence identity [26]. A default confidence threshold of 70% was used for taxonomic classification. Alpha and beta diversity analyses were performed using the QIIME 2 “diversity” plugin. Specifically, alpha diversity was quantified using Shannon’s index, Simpson’s index, Pielou’s evenness, and Faith’s phylogenetic diversity. Beta diversity was assessed by principal coordinate analysis (PCoA) of weighted and unweighted UniFrac distances. Phylogenetic analyses and figure generation were conducted in Python (v3.8.18) using standard bioinformatics libraries.

## 3. Results

### 3.1. Sequencing Workflow and Data Processing

**Figure 1:**
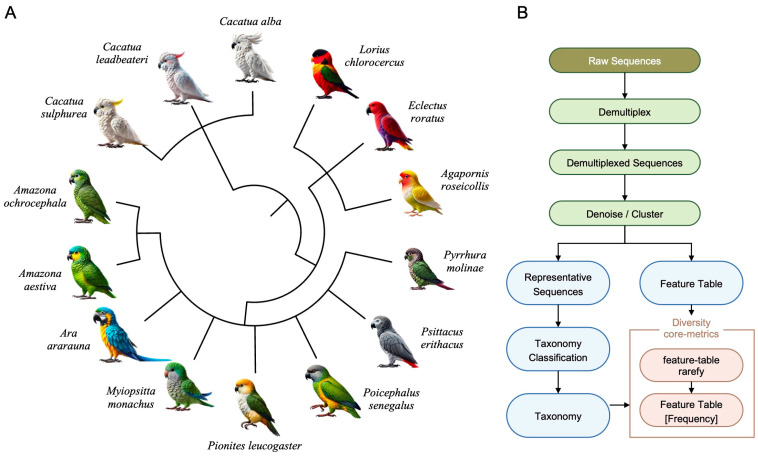
Phylogenetic overview of sampled parrot species and microbiome analysis workflow. (**A**) Phylogenetic tree of the 14 psittacine species examined in this study. (**B**) Workflow for 16S rRNA gene sequencing analysis. Raw reads were demultiplexed, denoised, and clustered to obtain representative amplicon sequence variants. Taxonomic classification was subsequently performed, and feature tables were generated for community composition and diversity analyses.

A phylogenetic tree covering 14 psittacine species representing diverse genera, including *Cacatua*, *Lorius*, *Eclectus*, *Agapornis*, *Psittacus*, *Poicephalus*, *Pionites*, *Myiopsitta*, *Ara*, and *Amazona* were constructed to provide an overview of the host species included in the present study (Figure 1A). This broad taxonomic representation captured the evolutionary variation across *Psittaciformes* and allowed assessment of whether host phylogeny influenced the gut microbial community structure. Sequencing data were processed using a standardized workflow to ensure comparability across samples (Figure 1B). Raw reads were demultiplexed, denoised, and clustered into amplicon sequence variants using the DADA2 algorithm implemented in QIIME 2 [24,25]. Representative sequences were taxonomically classified against the SILVA v132 database. Feature tables were generated and subsequently rarefied for downstream alpha and beta diversity analyses [26]. The processed data provided a basis for microbial community profiling and comparative assessment across host species. Quality control analysis confirmed that the sequencing process produced high-quality data suitable for downstream analysis. Most reads showed Phred quality scores of 30–40 (Supplementary Figure S1A), while the percentage of ambiguous bases (N) remained below 1% across the read lengths (Supplementary Figure S1B). Sequence count distributions varied among the samples (Supplementary Figure S1C,D), highlighting the necessity of normalization before comparative diversity analyses. Quality score distributions for both forward and reverse reads, assessed using a subset of 10,000 reads from 17,818,052 reads, further demonstrated high sequencing fidelity (Supplementary Figure S1E,F). Table 1 presents a detailed summary of sequencing performance across different parrot species.

### 3.2. Alpha Diversity Across Parrot Species

**Figure 2:**
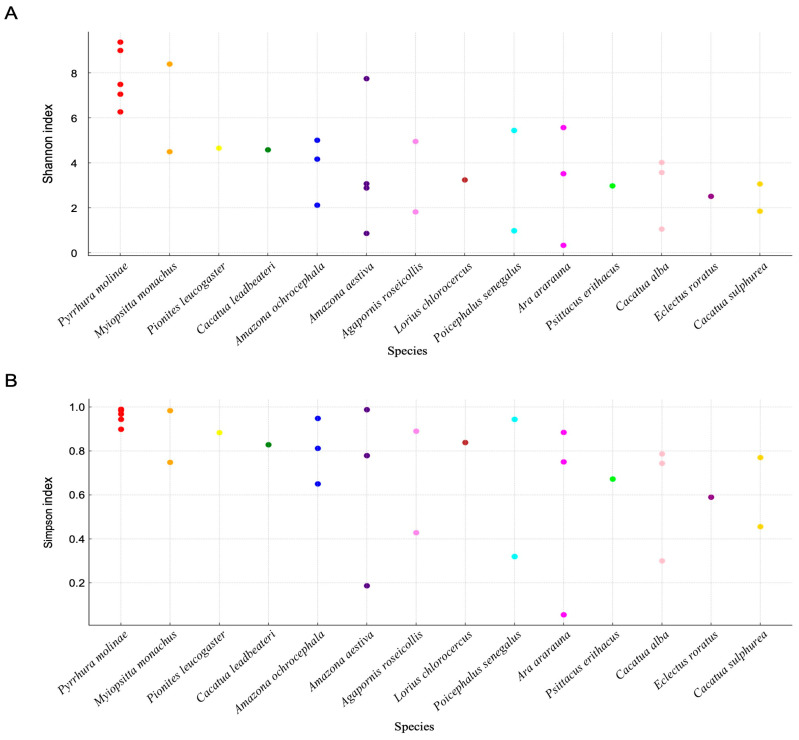
Alpha diversity of gut microbiota across 14 psittacine species. (**A**) Shannon and (**B**) Simpson indices for each parrot sample, grouped by host species. Each dot represents one individual. Higher index values indicate greater microbial richness and evenness.

Within-sample variation (alpha diversity) in gut microbial communities across the 14 parrot species was assessed using the Shannon and Simpson indices (Figure 2). Additional metrics included Pielou’s evenness and Faith’s phylogenetic diversity, as shown in Supplementary Figure S2. Considerable inter-individual variation was observed in most species, whereas overall diversity levels differed among hosts. *Pyrrhura molinae* and *Myiopsitta monachus* consistently exhibited high Shannon and Simpson index values, indicating richer and more evenly distributed microbial communities. *Cacatua alba* and *Cacatua sulphurea* showed the lowest diversity, reflecting reduced microbial richness and evenness. Other species, such as *Amazona aestiva* and *Agapornis roseicollis*, displayed intermediate diversity with pronounced individual variation. Within-species variability in alpha diversity was observed among species with multiple individuals. Shannon diversity ranged from minimum to maximum in species with ≥3 samples, consistent with inter-individual heterogeneity.

### 3.3. Beta Diversity and Community Structuring

**Figure 3:**
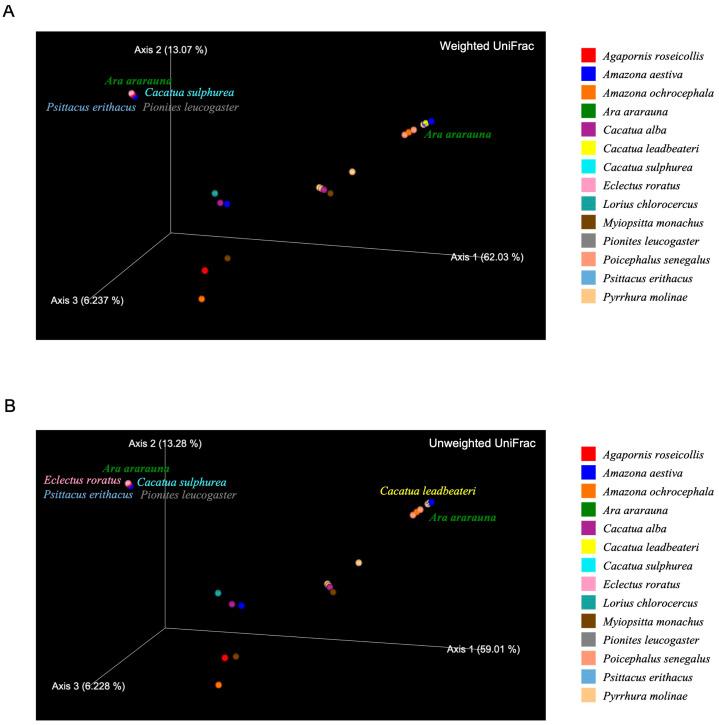
Beta diversity of gut microbiota across 14 psittacine species. Principal coordinate analysis plots based on (**A**) weighted and (**B**) unweighted UniFrac distances. Each point represents the gut microbial community of an individual parrot, color-coded by host species.

A PCoA based on weighted and unweighted UniFrac distances was conducted to further investigate the differences in gut microbial community composition among parrot species (Figure 3). Both analyses showed a clear separation of individuals along the primary coordinate axes, suggesting variation in community structure across species. The weighted UniFrac plot (Figure 3A)—which incorporates relative abundance information—revealed clustering of several *Amazona* and *Cacatua* species, indicating that these hosts shared similar dominant microbial taxa. *Agapornis roseicollis* and *Pyrrhura molinae* were positioned distantly from other species, reflecting distinct community structures. Contrastingly, the unweighted UniFrac plot (Figure 3B)—which accounts for the presence or absence of taxa alone—demonstrated similar overall clustering patterns, although with slightly greater dispersion among individuals. This finding indicates that while many core taxa are shared across species, rare lineages contribute to further differentiation. Within-species heterogeneity was also apparent in ordination space, as species represented by multiple individuals exhibited noticeable intra-species spread, consistent with inter-individual variation.

### 3.4. Microbial Community Composition

**Figure 4:**
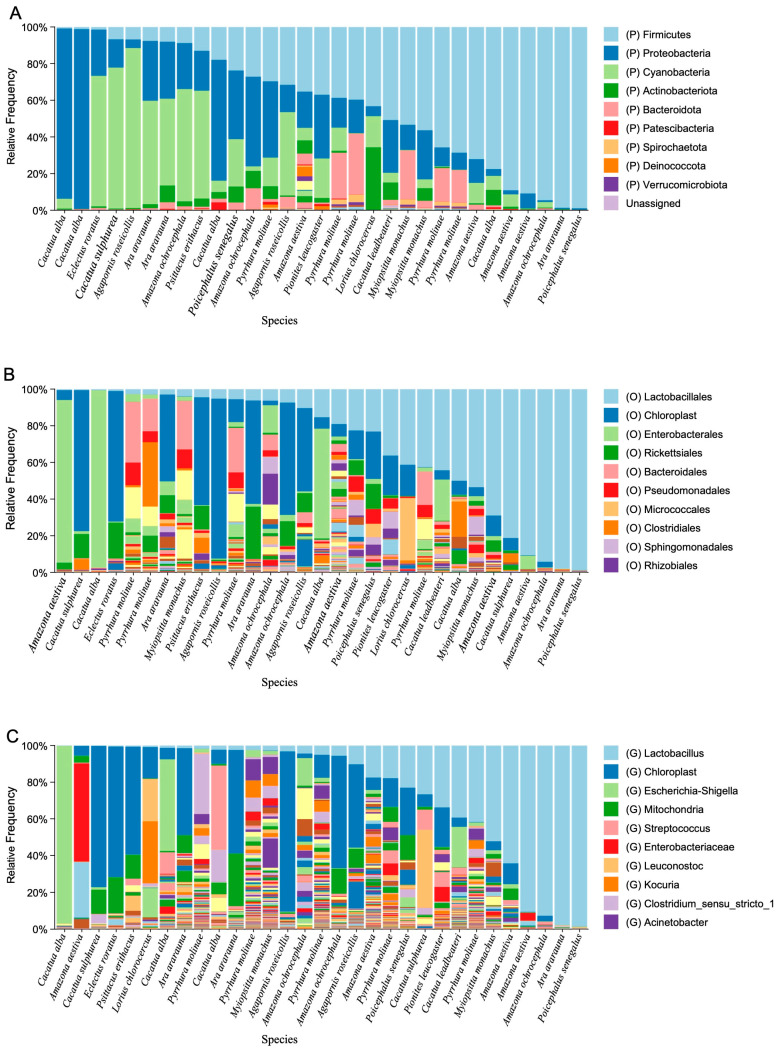
Taxonomic composition of gut microbiota in parrots at different levels. Stacked bar plots show the relative abundance of bacterial taxa across individual samples at the (**A**) phylum, (**B**) order, and (**C**) genus levels. The color legend presents the top 10 most abundant taxa, and the full list of detected taxa is available in the Supplementary Materials. P, phylum; O, order; G, genus.

Taxonomic profiling of the gut microbiota revealed distinct patterns of bacterial composition among individual parrots (Figure 4). Most samples were dominated by *Firmicutes* and *Proteobacteria* at the phylum level (Figure 4A), with additional contributions from *Cyanobacteria*, *Actinobacteria*, and *Bacteroidetes*. While *Firmicutes* accounted for the largest fraction in most individuals, certain samples exhibited marked enrichment of *Proteobacteria* or *Cyanobacteria*, indicating inter-individual variability. At the order level (Figure 4B), *Lactobacillales* was the most consistently abundant order, particularly in *Firmicutes*-dominated samples, highlighting the widespread presence of lactic acid bacteria. Other common orders included *Enterobacterales*, *Bacteroidales*, *Clostridiales*, and *Pseudomonadales*, although their relative abundance varied substantially among individuals. Genus-level analysis (Figure 4C) further confirmed the predominance of *Lactobacillus*, which was detected at high frequencies in nearly all host species. Additional genera such as *Escherichia–Shigella*, *Streptococcus*, *Enterobacteriaceae* (unclassified), and *Clostridium_sensu_stricto* were present in varying proportions, whereas taxa such as *Leuconostoc*, *Kocuria*, and *Acinetobacter* appeared sporadically.

### 3.5. Genus-Level Variation and Dietary Influence

**Figure 5:**
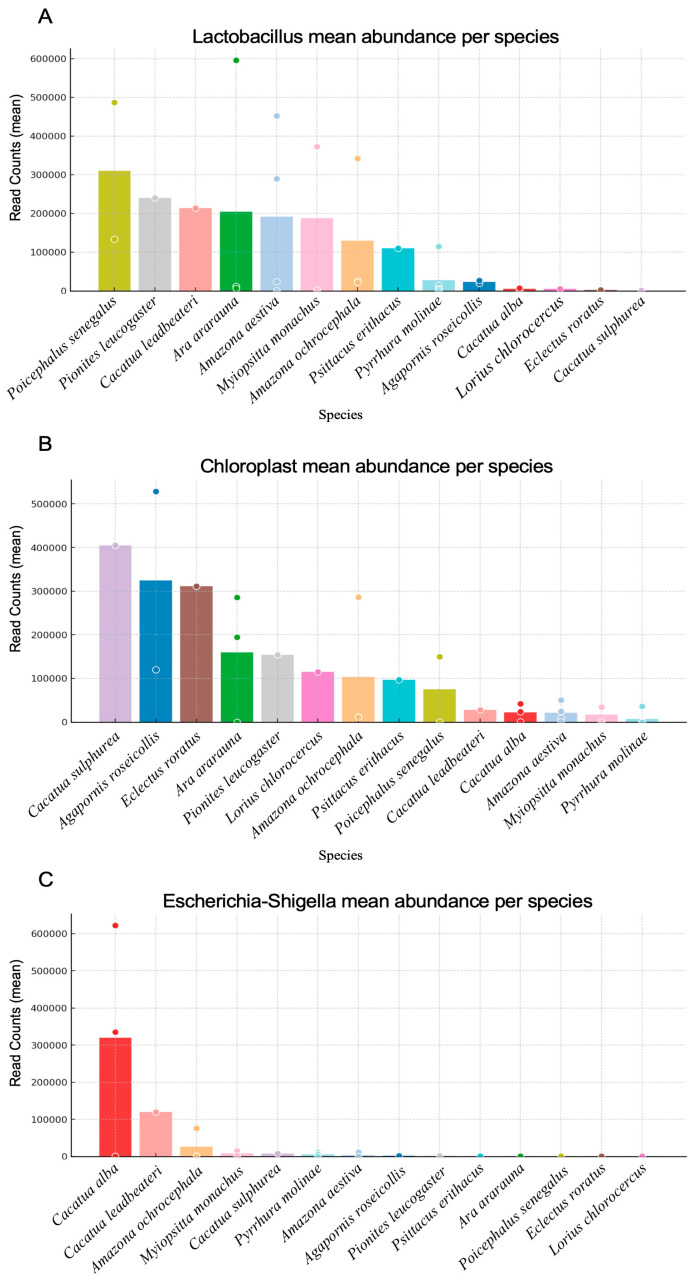
Host-specific microbial taxa patterns based on mean read counts. Bar plots show the mean read counts per species for (**A**) *Lactobacillus*, (**B**) chloroplast, and (**C**) *Escherichia–Shigella*. Bars represent species-level means, with individual data points overlaid.

The mean read counts of dominant genera were compared across parrot species to identify host-specific microbial taxa patterns (Figure 5). *Lactobacillus* was the most consistently enriched genus across all species (Figure 5A). The mean read counts were particularly high in *Poicephalus senegalus*, *Pionites leucogaster*, and *Cacatua leadbeateri*, whereas relatively lower counts were observed in cockatoos, such as *Cacatua alba* and *Cacatua sulphurea*. These results support the designation of *Lactobacillus* as a core taxon within the gut microbiome of parrots, with interspecies differences in overall abundance. Chloroplast-assigned reads showed higher mean read counts in *Cacatua sulphurea*, *Agapornis roseicollis*, and *Eclectus roratus*, whereas the other species showed only moderate to low read counts (Figure 5B). *Escherichia–Shigella* displayed restricted but strongly elevated read counts in specific hosts (Figure 5C).

## 4. Discussion

To our knowledge, this study represents one of the most extensive comparative surveys of gut microbiota across parrots, encompassing 14 species and 31 individuals sampled from privately owned companion birds. The study aimed to provide exploratory baseline data for companion parrots maintained under comparable conditions, while the number of individuals per species was limited and uneven. In this context, our findings extend previous studies that examined fewer species or individuals and contribute to a growing body of knowledge on the gut microbiota of parrots.

Alpha diversity analyses revealed marked variability both within and among species. *Psittacus erithacus* and *Eclectus roratus* exhibited relatively high richness and evenness, whereas some *Cacatua* species displayed lower values. No consistent species-specific patterns were detected under the present sampling design, suggesting that microbial richness in parrots is not strongly constrained by host phylogeny. This differs from patterns observed in mammals, where microbial diversity frequently mirrors host relatedness [27,28,29], underscoring the need to consider alternative structuring factors in avian systems. The substantial inter-individual variation within species further supports the role of environmental exposure in influencing microbial community composition.

Across all samples, community composition was dominated by *Firmicutes* and *Proteobacteria*, aligning with earlier reports on parrots and other birds such as poultry and passerines [13,16,30]. The consistent detection of *Lactobacillus* across nearly all individuals suggests that this genus represents part of a core microbiota in parrots. This inference is supported by its frequent occurrence and well-documented roles in carbohydrate metabolism, plant-derived substrate fermentation, and gut homeostasis maintenance in other vertebrates [10,16]. The pronounced enrichment of *Lactobacillus* in nectarivorous parrots and the higher abundance of *Escherichia–Shigella* and *Streptococcus* in seed-eating species suggest that feeding ecology is a key determinant of microbial community assembly in parrots [31]. However, functional roles cannot be directly inferred from 16S rRNA gene sequencing data, and interpretations regarding metabolic or health-related functions remain speculative and rely on the prior literature. Future studies integrating shotgun metagenomics or metabolomics are required to validate these functional hypotheses. In contrast, the presence of opportunistic taxa, such as *Escherichia–Shigella* and *Streptococcus*, in certain individuals indicates that microbial composition can shift considerably depending on dietary context or host condition. Chloroplast-assigned reads likely reflect dietary plant materials. Their uneven distribution among species further highlights the influence of dietary inputs on microbial profiles.

Beta diversity analyses further demonstrated that microbial communities did not segregate according to host taxonomy. Instead, extensive overlap was observed among individuals across species, indicating weak phylogenetic structuring of the gut microbiota. Comparisons with studies of wild psittacines provide important context for interpreting these patterns. For instance, the cloacal microbiota of wild parrots in good health differed from that of captive parrots, as shown by UniFrac-based separation between groups and the enrichment of specific taxa, such as *Staphylococcus saprophyticus* in wild birds and *Escherichia coli* in captive birds [14]. Research on the critically endangered Kakapo (*Strigops habroptilus*) also revealed a gastrointestinal microbiota with relatively low diversity, predominantly consisting of *Firmicutes* (including lactic acid bacteria) and *Gammaproteobacteria* [32]. Longitudinal sampling of chick feces demonstrated frequent dominance by *Escherichia–Shigella* [32]. These findings align with observations in other captive vertebrates, where husbandry and feeding regimes exert stronger influences on microbiota than host evolutionary history [33,34]. Beyond their ecological relevance, these results may also inform local veterinary surveillance by providing baseline gut microbiota references for clinically healthy companion parrots, with potential relevance to One Health monitoring in captive settings.

This study has some limitations. First, host-related factors such as age, sex, and precise health status—although all individuals appeared clinically healthy upon visual inspection—could not be quantitatively incorporated because of incomplete or heterogeneous metadata. Additionally, the limited and uneven number of individuals per species constrained the ability to disentangle the relative contributions of host-related factors and detect species-specific patterns in microbiome variation. As all individuals were sampled at a single time point, this study provides only a snapshot of gut microbiota composition and does not permit assessment of temporal stability, causal relationships, or responses to dietary or environmental changes. Therefore, longitudinal sampling will be required to investigate microbiome dynamics over time.

## 5. Conclusions

This study revealed that *Firmicutes* and *Proteobacteria* predominated the gut microbiota of companion parrots, accompanied by substantial inter-individual variation. Feeding ecology emerged as an important structuring factor. Overall, these findings provide exploratory baseline insights into gut microbiome variation in captive parrots and establish a framework for integrating microbiome data into aviculture and conservation management. Particularly, microbiome-informed dietary strategies and probiotic interventions may represent promising avenues for improving the health and management of captive parrot populations.

## Figures and Tables

**Table 1:** Sequencing data summary for parrot samples. Quality specification required that ≥80% of bases had a phred quality score of at least Q30 for 2 × 300 bp paired-end reads.

Group	Sample	Read Pairs	Yield (bp)	% ≥Q30 Bases	Mean Quality Score
*Agapornis roseicollis*	B1	710,842	426,505,200	85.7	Q33.4
*Agapornis roseicollis*	B2	339,566	203,739,600	87.2	Q33.7
*Amazona aestiva*	B3	570,814	342,488,400	85.3	Q33.3
*Amazona aestiva*	B4	165,809	99,485,400	85.4	Q33.3
*Amazona aestiva*	B5	527,891	316,734,600	85.2	Q33.2
*Amazona aestiva*	B6	579,258	347,554,800	85.1	Q33.2
*Amazona ochrocephala*	B7	577,411	346,446,600	85.8	Q33.4
*Amazona ochrocephala*	B8	528,603	317,161,800	84.2	Q33.0
*Amazona ochrocephala*	B9	627,090	376,254,000	84.3	Q33.0
*Ara ararauna*	B10	696,473	417,883,800	84.9	Q33.1
*Ara ararauna*	B11	674,762	404,857,200	85.7	Q33.4
*Ara ararauna*	B12	557,047	334,228,200	85.3	Q33.3
*Cacatua alba*	B13	781,650	468,990,000	84.9	Q33.2
*Cacatua alba*	B14	820,886	492,531,600	85.6	Q33.3
*Cacatua alba*	B15	381,556	228,933,600	87	Q33.6
*Cacatua leadbeateri*	B16	647,626	388,575,600	85.2	Q33.2
*Cacatua sulphurea*	B17	638,143	382,885,800	85.5	Q33.3
*Cacatua sulphurea*	B18	309,699	185,819,400	86.8	Q33.5
*Eclectus roratus*	B19	574,662	344,797,200	84.2	Q33.0
*Lorius chlorocercus*	B20	876,649	525,989,400	85.2	Q33.3
*Myiopsitta monachus*	B21	850,967	510,580,200	85.3	Q33.3
*Myiopsitta monachus*	B22	147,880	88,728,000	83.1	Q32.6
*Pionites leucogaster*	B23	856,452	513,871,200	85.4	Q33.3
*Poicephalus senegalus*	B24	818,577	491,146,200	85.2	Q33.3
*Poicephalus senegalus*	B25	579,247	347,548,200	85	Q33.2
*Psittacus erithacus*	B26	716,427	429,856,200	85.8	Q33.4
*Pyrrhura molinae*	B27	772,022	463,213,200	85.7	Q33.3
*Pyrrhura molinae*	B28	396,145	237,687,000	87.5	Q33.8
*Pyrrhura molinae*	B29	388,770	233,262,000	87.7	Q33.8
*Pyrrhura molinae*	B30	380,060	228,036,000	86.7	Q33.5
*Pyrrhura molinae*	B31	325,068	195,040,800	87.2	Q33.7

## Data Availability

The raw FASTQ files for the metagenomic sequencing data can be accessed from the NCBI Sequence Read Archive (SRA) under BioProject ID PRJNA1128245. These files are referenced by the accession identifiers provided in Supplementary Table S1. This project was titled “Characterization and Comparative Analysis of Gut Microbiomes Across Fourteen Parrot Species”.

## Supplementary Materials

The following supporting information can be downloaded at: https://www.mdpi.com/article/10.3390/vetsci13020185/s1, Figure S1: Summary of sequence quality profiles and read distributions across samples; Table S1: NCBI Accession column for PRJNA1128245; Figure S2: Pielou’s evenness and Faith’s phylogenetic diversity indices of gut microbiota across 14 psittacine species; Supplementary Materials: Full list of detected taxa for Figure 4.

## Abbreviations

The following abbreviations are used in this manuscript:

DNB	DNA nanoballs
PCR	Polymerase chain reaction
PCoA	Principal coordinate analysis
