# Supplementary material for: Characterization and Comparative Analysis of Gut Microbiomes in Fourteen Parrot Species
Source: Vet Sci. 2026 Feb 12;13(2):185. doi: 10.3390/vetsci13020185 (PMC12944863; doi:10.3390/vetsci13020185)
Supplement: Supplementary file 1 [file vetsci-13-00185-s001.zip › Supplementary TableS1.pdf]

Supplementary Table S1. NCBI Accession column for PRJNA1128245

Run	BioSample	Bases	Bytes	Experiment	Host	Sample Name
SRR29547976	SAMN42042499	417.88 M	309.33 Mb	SRX25055233	Ara ararauna	B10
SRR29547977	SAMN42042498	376.25 M	280.56 Mb	SRX25055232	Amazona ochrocephala	B9
SRR29547978	SAMN42042497	317.16 M	236.24 Mb	SRX25055231	Amazona ochrocephala	B8
SRR29547979	SAMN42042496	346.45 M	254.73 Mb	SRX25055230	Amazona ochrocephala	B7
SRR29547980	SAMN42042495	347.55 M	256.97 Mb	SRX25055229	Amazona aestiva	B6
SRR29547981	SAMN42042494	316.73 M	234.16 Mb	SRX25055228	Amazona aestiva	B5
SRR29547982	SAMN42042520	195.04 M	141.96 Mb	SRX25055227	Pyrrhura molinae	B31
SRR29547983	SAMN42042493	99.49 M	73.80 Mb	SRX25055226	Amazona aestiva	B4
SRR29547984	SAMN42042519	228.04 M	166.70 Mb	SRX25055225	Pyrrhura molinae	B30
SRR29547985	SAMN42042518	233.26 M	168.74 Mb	SRX25055224	Pyrrhura molinae	B29
SRR29547986	SAMN42042517	237.69 M	172.30 Mb	SRX25055223	Pyrrhura molinae	B28
SRR29547987	SAMN42042516	463.21 M	340.70 Mb	SRX25055222	Pyrrhura molinae	B27
SRR29547988	SAMN42042515	429.86 M	316.01 Mb	SRX25055221	Psittacus erithacus	B26
SRR29547989	SAMN42042514	347.55 M	257.28 Mb	SRX25055220	Poicephalus senegalus	B25
SRR29547990	SAMN42042513	491.15 M	363.08 Mb	SRX25055219	Poicephalus senegalus	B24
SRR29547991	SAMN42042512	513.87 M	378.71 Mb	SRX25055218	Pionites leucogaster	B23
SRR29547992	SAMN42042511	88.73 M	67.18 Mb	SRX25055217	Myiopsitta monachus	B22
SRR29547993	SAMN42042510	510.58 M	376.71 Mb	SRX25055216	Myiopsitta monachus	B21
SRR29547994	SAMN42042492	342.49 M	252.87 Mb	SRX25055215	Amazona aestiva	B3
SRR29547995	SAMN42042509	525.99 M	388.61 Mb	SRX25055214	Lorius chlorocercus	B20
SRR29547996	SAMN42042508	344.80 M	257.14 Mb	SRX25055213	Eclectus roratus	B19
SRR29547997	SAMN42042507	185.82 M	135.70 Mb	SRX25055212	Cacatua sulphurea	B18
SRR29547998	SAMN42042506	382.89 M	282.50 Mb	SRX25055211	Cacatua sulphurea	B17
SRR29547999	SAMN42042505	388.58 M	287.20 Mb	SRX25055210	Lophochroa leadbeateri	B16
SRR29548000	SAMN42042504	228.93 M	166.89 Mb	SRX25055209	Cacatua alba	B15
SRR29548001	SAMN42042503	492.53 M	362.57 Mb	SRX25055208	Cacatua alba	B14

SRR29548002	SAMN42042502	468.99 M	346.86 Mb	SRX25055207	Cacatua alba	B13
SRR29548003	SAMN42042501	334.23 M	246.83 Mb	SRX25055206	Ara ararauna	B12
SRR29548004	SAMN42042500	404.86 M	297.94 Mb	SRX25055205	Ara ararauna	B11
SRR29548005	SAMN42042491	203.74 M	148.25 Mb	SRX25055204	Agapornis roseicollis	B2
SRR29548006	SAMN42042490	426.51 M	313.84 Mb	SRX25055203	Agapornis roseicollis	B1
