# Supplementary figures and images for: Characterization and Comparative Analysis of Gut Microbiomes in Fourteen Parrot Species
Source: Vet Sci. 2026 Feb 12;13(2):185. doi: 10.3390/vetsci13020185 (PMC12944863; doi:10.3390/vetsci13020185)

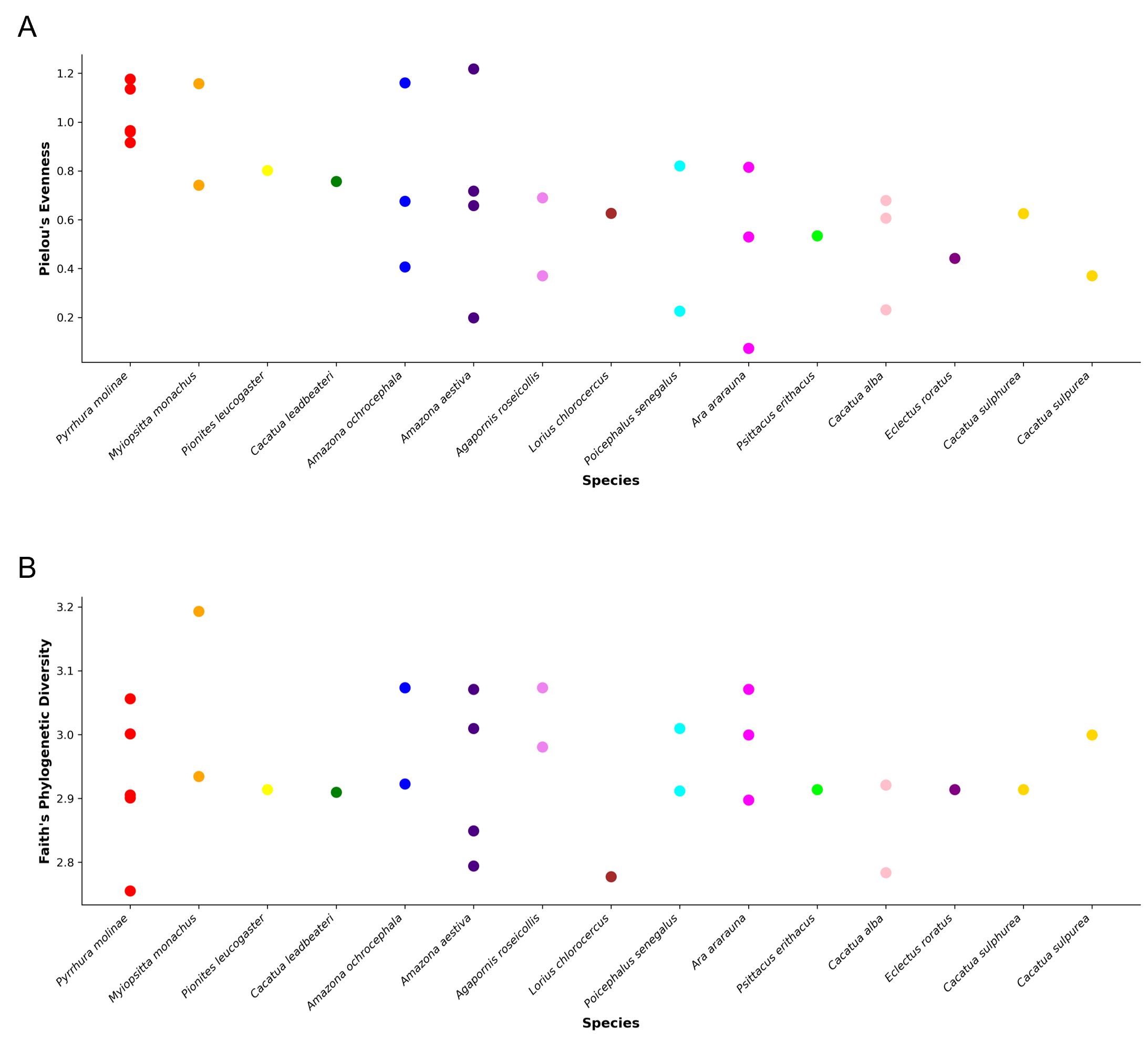

Supplement: Supplementary file 1 [file vetsci-13-00185-s001.zip › Supplementary Figure S2.png]

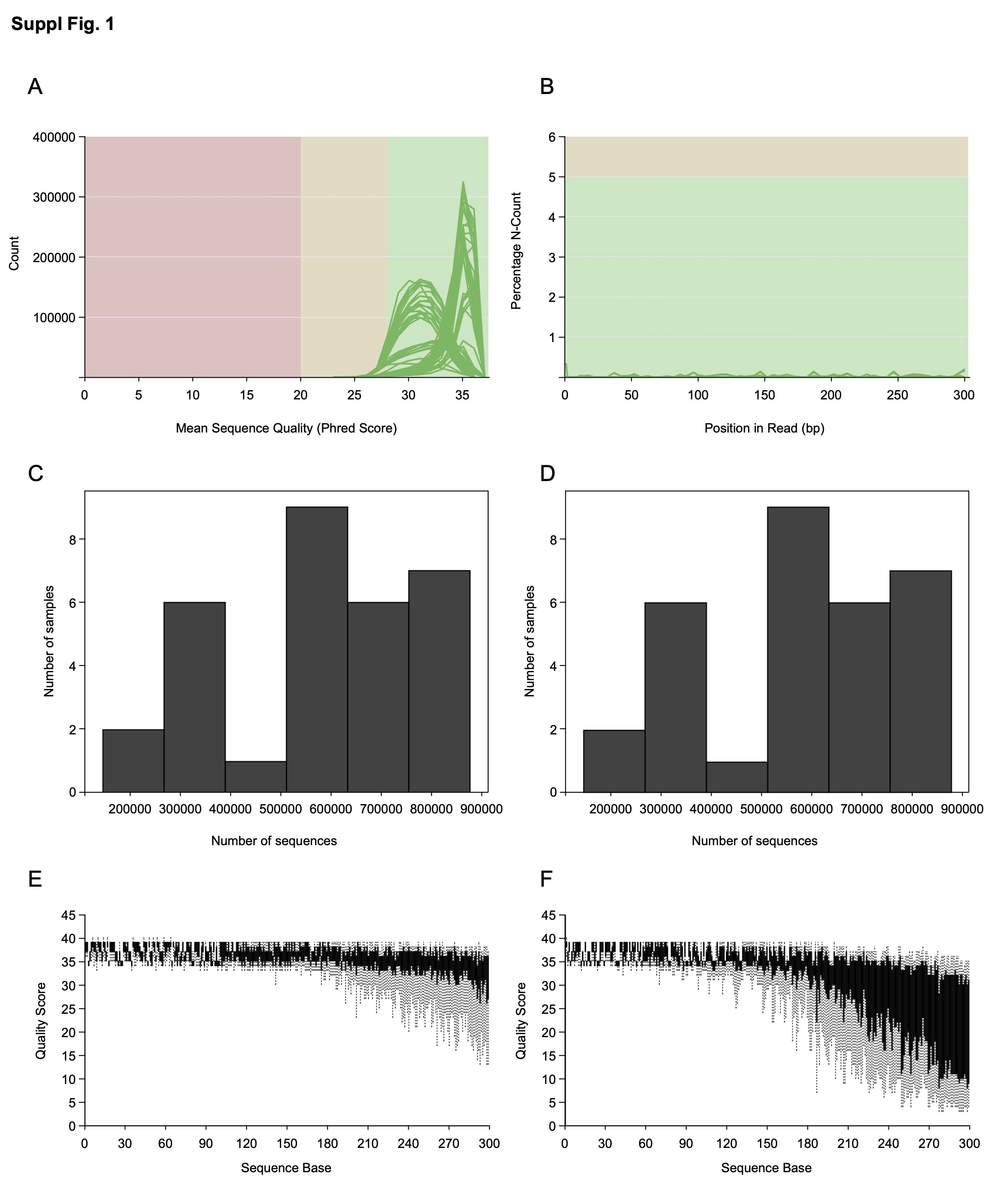

Supplement: Supplementary file 1 [file vetsci-13-00185-s001.zip › Supplementary Figure S1.jpg]
